# Fast parallel image registration on CPU and GPU for diagnostic classification of Alzheimer's disease

**DOI:** 10.3389/fninf.2013.00050

**Published:** 2014-01-16

**Authors:** Denis P. Shamonin, Esther E. Bron, Boudewijn P. F. Lelieveldt, Marion Smits, Stefan Klein, Marius Staring, for the Alzheimer's Disease Neuroimaging Initiative

**Affiliations:** ^1^Division of Image Processing (LKEB), Department of Radiology, Leiden University Medical CenterLeiden, Netherlands; ^2^Biomedical Imaging Group Rotterdam, Departments of Medical Informatics and RadiologyErasmus MC, Rotterdam, Netherlands; ^3^Intelligent Systems Group, Faculty of EEMCS, Delft University of TechnologyDelft, Netherlands; ^4^Department of RadiologyErasmus MC, Rotterdam, Netherlands

**Keywords:** image registration, parallelization, acceleration, OpenCL, elastix, Alzheimer's disease

## Abstract

Nonrigid image registration is an important, but time-consuming task in medical image analysis. In typical neuroimaging studies, multiple image registrations are performed, i.e., for atlas-based segmentation or template construction. Faster image registration routines would therefore be beneficial. In this paper we explore acceleration of the image registration package elastix by a combination of several techniques: (i) parallelization on the CPU, to speed up the cost function derivative calculation; (ii) parallelization on the GPU building on and extending the OpenCL framework from ITKv4, to speed up the Gaussian pyramid computation and the image resampling step; (iii) exploitation of certain properties of the B-spline transformation model; (iv) further software optimizations. The accelerated registration tool is employed in a study on diagnostic classification of Alzheimer's disease and cognitively normal controls based on T1-weighted MRI. We selected 299 participants from the publicly available Alzheimer's Disease Neuroimaging Initiative database. Classification is performed with a support vector machine based on gray matter volumes as a marker for atrophy. We evaluated two types of strategies (voxel-wise and region-wise) that heavily rely on nonrigid image registration. Parallelization and optimization resulted in an acceleration factor of 4–5x on an 8-core machine. Using OpenCL a speedup factor of 2 was realized for computation of the Gaussian pyramids, and 15–60 for the resampling step, for larger images. The voxel-wise and the region-wise classification methods had an area under the receiver operator characteristic curve of 88 and 90%, respectively, both for standard and accelerated registration. We conclude that the image registration package elastix was substantially accelerated, with nearly identical results to the non-optimized version. The new functionality will become available in the next release of elastix as open source under the BSD license.

## 1. Introduction

Image registration is a frequently used technique in medical image processing. It refers to the process of automatically aligning imaging data, where a *moving (target) image I_M_* is deformed to mimick a *fixed (reference) image I_F_*. In other words, registration is the problem of finding a coordinate transformation ***T*** that makes *I_M_*(***T***) spatially aligned with *I_F_*. The quality of alignment is defined by a cost function 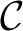. The optimal coordinate transformation is estimated by minimizing the cost function with respect to ***T***, usually by means of an iterative optimization method embedded in a hierarchical (multiresolution) scheme. Extensive reviews on the subject of image registration are given in Brown (1992); Maintz and Viergever (1998). Areas of application include the alignment of data sets from different modalities (Mattes et al., 2003) to fuse information, comparison of follow-up with baseline scans (Staring et al., 2007) to follow disease development, alignment of different MR sequences for extraction of quantitative MR parameters such as in diffusion tensor imaging or MR relaxometry (Alexander et al., 2001; Bron et al., 2013), alignment of pre- and post-contrast images (Rueckert et al., 1999) to aid breast cancer detection and diagnosis, and updating treatment plans for radiotherapy and surgery (Pennec et al., 2003).

Accordingly, most neuroimaging research also requires image registration. Registration is mainly needed to create a reference frame, which enables comparison between subjects, between image sequences and over time. This reference framework can either be a common template space to which every subject's image is registered (Mazziotta et al., 1995; Seghers et al., 2004; Ashburner, 2007), or a region-labeling system for example obtained with multi-atlas segmentation (Heckemann et al., 2006). Many different neuroimaging applications rely on such a reference framework: statistical group comparisons (Friston et al., 1994), voxel-based morphometry (Ashburner and Friston, 2000), tissue segmentation (Fischl et al., 2002; Ashburner and Friston, 2005), and diagnostic classification (Klöppel et al., 2008; Magnin et al., 2009; Cuingnet et al., 2011). In these applications, registration methods are used to align the data with the reference frame.

To create a reference frame that maps between different subjects, nonrigid image registration is applied, which can be very time-consuming. Runtime depends on the specific cost function, transformation complexity, data size, and optimization strategy. The first three items have increased in complexity over the years: more complex cost functions were needed for multi-modal image registration (Maes et al., 1997), nonrigid transformations have many parameters frequently generating a 10^6^ dimensional space to be optimized, and data sizes have increased tremendously with the advent of new scanners. This results in a typical runtime of registration algorithms in the order of at best 15 min, up to hours (Klein et al., 2009a); future acquisition-side improvements in image resolution may even increase that number. Moreover, for creating a reference frame, many registrations are required: every subject needs to be aligned with the template space, or, when using multi-atlas segmentation, every atlas image needs to be aligned with every subject image.

One of the neuroimaging applications mentioned above is diagnostic classification. As the incidence of Alzheimer's Disease (AD) as well as the need for early and accurate diagnosis is dramatically growing (Alzheimer's Association, 2012), automated classification is an emerging research field. To advance the diagnosis of AD in individual patients, machine-learning techniques can be applied to imaging or other data. These techniques use labeled data to train a classifier to categorize two groups (e.g., patients and controls). Several studies demonstrated the successful classification of dementia based on atrophy using such machine-learning methods (e.g., Fan et al., 2008; Klöppel et al., 2008; Vemuri et al., 2008; Magnin et al., 2009; Cuingnet et al., 2011; Koikkalainen et al., 2012). The atrophy features used in these studies are derived from structural MR using two main approaches: voxel-wise (e.g., Klöppel et al., 2008) and region-wise (e.g., Magnin et al., 2009) feature extraction. Voxel-wise methods use a feature for each voxel in the brain, for example the gray matter (GM) density as an atrophy measure. In the region-wise approach, a region-labeling consisting of a set of brain regions is used to calculate a feature, for example the GM volume in each region of interest (ROI). Both approaches require many nonrigid image registrations: in the voxel-wise approach, to align all scans in a template space, and in the region-wise approach, to obtain a region-labeling for each individual scan using multi-atlas segmentation.

In this paper we explore the acceleration of image registration in the context of neuroimaging applications, by a combination of methods. Critical registration components are parallelized, utilizing the CPU as well as the GPU, certain properties of the B-spline transformation model are exploited, and source code is optimized. These efforts are integrated in the popular open source registration toolkit elastix (Klein et al., 2010), which is based on the Insight ToolKit (ITK, (Ibánez et al., 2005)). elastix aims to deliver convenient access to a wide range of image registration algorithms to end-users (researchers as well as medical practitioners). For the GPU implementation, the recently introduced OpenCL functionality in ITKv4 was improved, extended and exploited. The developed functionality will become available in the next release of elastix, as open source under the BSD license.

Others have also addressed registration performance by means of parallel processing. An overview of both CPU and GPU work is given by Shams et al. (2010b). Many authors use derivative-free optimization techniques, and therefore focus on low dimensional transformations, on a cluster of computers (Warfield et al., 1998), using a GPU (Shams et al., 2010a) or an FPGA (Castro-Pareja et al., 2003). Rohlfing and Maurer (2003) proposed a scheme for nonrigid registration using finite differences for the derivative computation, distributing the elements of the derivative over the processing elements. Results were evaluated by visual inspection. Saxena et al. (2010) implemented an analytical derivative based nonrigid registration scheme on the GPU for mutual information, using CUDA. In this paper we present methods that (i) exploit both the CPU and hardware accelerators (GPU, and potentially also the FPGA), (ii) do not require a cluster of computers but runs on a single computer, (iii) are based on the analytical cost function derivative, enabling gradient based (stochastic) optimization, (iv) work for 2D and 3D image registration, implemented for various metrics and various transformation types, (v) will be made freely available, and (vi) are quantitatively validated to obtain similar results as the unoptimized registration method.

The paper is outlined as follows. In Section 2 preliminary information is given about image registration, elastix, OpenCL and ITK. The registration accelerations are described in Section 3, together with the methodology for voxel-wise and region-wise diagnostic classification of AD. Experiments and results are given in Section 4, detailing the obtained speedup factors (Section 4.2 and 4.3). In Section 4.4 an accuracy analysis is made comparing original and optimized versions of elastix. For this evaluation, we used structural MR data of AD patients and healthy volunteers from the Alzheimer's Disease Neuroimaging Initiative (ADNI) database. The paper is concluded in Section 5.

## 2. Preliminaries

### 2.1. Image registration

Image registration is the process of aligning images, and can be defined as an optimization problem:


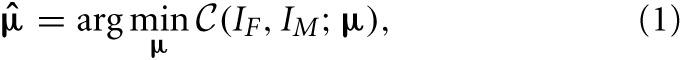


with *I_F_*(***x***): ***x*** ∈ Ω_*F*_ → ℝ and *I_M_*(***x***): ***x*** ∈ Ω_*M*_ → ℝ the *d*-dimensional fixed and moving image, respectively, on their domains Ω_*F*_ and Ω_*M*_, and **μ** the vector of parameters of size *N* that model the transformation ***T***_μ_. The cost function 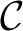 consists of a similarity measure 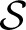
(*I*_*F*_, *I_M_*; **μ**) that defines the quality of alignment, and optionally a regularizer. Examples of the first are the mean square difference (MSD), normalized correlation (NC), and mutual information (MI) (Maes et al., 1997) measure; examples of the last are the bending energy (Rueckert et al., 1999) and rigidity penalty term (Staring et al., 2007). Optimization is frequently performed using a form of gradient descent:


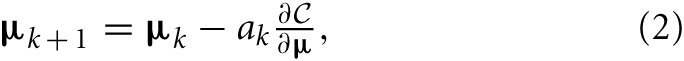


with *a_k_* the step size at iteration *k*. The derivative of the cost function can commonly be written as


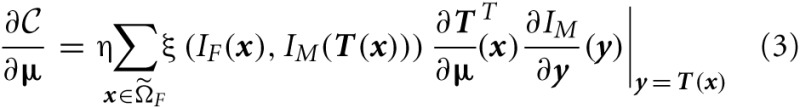


with ξ(·) a continuous function mapping to ℝ, $\tilde{\Omega }$*_F_* a discrete set of coordinates from Ω*_F_*, and η = 1 / |$\tilde{\Omega }$*_F_*| a normalization factor. For the MSD metric for example we have ξ(·) = *I_F_*(***x***) − *I_M_*(***T***(***x***)). This form holds for all the above mentioned similarity metrics, while for regularizers a similar form can be derived. In this paper we focus on stochastic optimization methods (Klein et al., 2007), where the derivative is computed with a small number |$\tilde{\Omega }$*_F_*| of randomly drawn samples, newly selected in each iteration *k*. Specifically, we use the adaptive stochastic gradient descent optimizer (Klein et al., 2009b), which automatically computes the step size *a_k_*. The computation time of this step is addressed in other work (Qiao et al., 2014).

Image registration is usually embedded in a multi-resolution framework, and after the optimization procedure (1) has finished, a resampling of the moving image is desired to generate the registration result $I_{M}(T_{\hat{\mu }})$.

### 2.2. GPUs and OpenCL

Multi-core computers have enabled the acceleration of a wide variety of computationally intensive applications. Nowadays, another type of hardware promises even higher computational performance: the graphics processing unit (GPU), which has a highly parallel hardware structure. This makes them more effective than general purpose CPUs for algorithms where processing of large blocks of data can be performed in parallel. The increasing computing power of GPUs gives them considerably higher peak computing power than CPUs. For example, NVidia's GeForce GTX 780 GPU provides 3977 Gflop/s and AMDs HD7970 GPU 3788 Gflop/s, while Intels Xeon X5675 CPU reaches only 144 Gflop/s.

Writing parallel programs to take full advantage of this GPU power is still a challenge. The OpenCL C programming language (www.khronos.org/opencl/) can be used to create programs that can be executed on one or more heterogeneous devices such as CPUs, GPUs, FPGAs and potentially other devices developed in the future. CUDA (www.nvidia.com/object/cuda_home_new.html) on the other hand is NVidia's C language targeted to NVidia GPUs only. OpenCL is maintained by the non-profit technology consortium Khronos Group. An OpenCL program is similar to a dynamic library, and an OpenCL kernel is similar to an exported function from the dynamic library. In OpenCL programmers can use OpenCL command queue execution and events to explicitly specify runtime dependencies between arbitrary queued commands, which is different from C(++) where sequential execution of commands is always implied. OpenCL is based on the C99 language specification with some restrictions and specific extensions to the language for parallelism.

In this project we decided to adopt OpenCL for algorithm implementation for two reasons: (i) OpenCL solutions are independent of the GPU hardware vendor, and can even be run on other hardware accelerators, thereby broadening the applicability of this work; (ii) Our image registration package elastix is largely based on the Insight Toolkit (ITK), in which OpenCL also was adopted recently.

### 2.3. elastix and ITKv4

Parallelization is performed in the context of the image registration software elastix (Klein et al., 2010), available at http://elastix.isi.uu.nl. The software is distributed as open source via periodic software releases under a BSD license. The software consists of a collection of algorithms that are commonly used to solve (medical) image registration problems. The modular design of elastix allows the user to quickly configure, test, and compare different registration methods for a specific application. A command-line interface enables automated processing of large numbers of data sets, by means of scripting.

elastix is based on the well-known open source Insight Segmentation and Registration Toolkit (ITK) (Ibánez et al., 2005) available at www.itk.org. This library contains a lot of image processing functionality, and delivers an extremely well tested coding framework. The ITK is implemented in C++, nightly tested, has a rigorous collaboration process, and works on many platforms and compilers. The use of the ITK in elastix implies that the low-level functionality (image classes, memory allocation, etc.) is thoroughly tested. Naturally, all image formats supported by the ITK are supported by elastix as well. elastix can be compiled on multiple operating systems (Windows, Linux, Mac OS X), using various compilers (MS Visual Studio, Clang, GCC), and supports both 32 and 64 bit systems.

## 3. Methods

As described in Section 2.1 the image registration algorithm consists of multiple parts: general tasks such as image reading and setting up the registration pipeline, pyramid construction, then iteratively derivative computation and updating of the parameter vector using (2), and finally resampling. To accelerate the registration algorithm, we identified the pyramid construction, the optimization routine and the resampling step as the most dominant parts in terms of performance. Acceleration possibilities for the optimization routine are identified by recognizing parallelization options, by manual inspection of the source code, and by the use of the Callgrind profiling tool (Weidendorfer et al., 2004), see Section 3.1. This component of the registration algorithm is performed on the CPU. Both pyramid construction and resampling are in this work off-loaded to the GPU, because these components exhibit clear opportunities for massive data parallelization, see Section 3.2. Finally, in Section 3.3, we present the methods used for validation of the optimized registration procedure with an experiment on diagnostic classification of AD which heavily relies on image registration.

### 3.1. CPU

Considering Equation (3) we see that image registration constitutes a loop over the image samples as a key component of the algorithm. This part can be computed in parallel by distributing the image samples in $\tilde{\Omega }$*_F_* over different threads. This is implemented by a fork-and-join model using the thread system of the ITK: in each iteration *T* threads are created (forking), *T* derivatives ***g**^t^_k_* = ∂
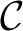
*^t^* / ∂**μ** over the sample subsets are computed in parallel (*t* denoting the thread id), and the results are joined into a single derivative. Functions that are used by the different threads were made thread-safe, and preparation functionality was refactored and called only once by the master thread. Where possible, we avoided false sharing of data (Bolosky and Scott, 1993), which can substantially affect performance. This recipe was implemented in elastix for several similarity measures (MSD, NC, MI, kappa statistic), and the bending energy penalty term.

Parallel computation was also implemented at several other places, namely for aggregation of the thread derivatives ***g**^t^_k_* to a single derivative ***g**_k_*, and for performing the update step of the optimizer, see Equation (2). At these places some straightforward vector arithmetic is performed on ***g**_k_* and **μ***_k_*, which are vectors of possibly very long size (up to 10^6^). Parallelization can be performed here by threads working on disjoint parts of the vectors. Implementations using the ITK thread model and OpenMP were created.

Again considering Equation (3) we can see that part of the computation is in calculating ∂ ***T*** / ∂**μ** ≐ ***J***. The Callgrind profiler confirmed this as a performance bottleneck. For the general case the matrix ***J*** has size *d* × *N, N* being the size of **μ**. In case of a B-spline transformation however, this matrix is mostly empty due to the compact support of the B-spline basis function, resulting in a matrix of size *d* × *dP, P* = (*O* + 1)*^d^* « *N*, with *O* the B-spline order (usually equal to 3). This much smaller matrix has the form:

(4) $$J(x)≐\frac{\partial T}{\partial \mu }(x)\equiv \left[\begin{array}{ccc} j_{1}\cdots j_{P} & 0\cdots 0 & 0\cdots 0\\ 0\cdots 0 & j_{1}\cdots j_{P} & 0\cdots 0\\ 0\cdots 0 & 0\cdots 0 & j_{1}\cdots j_{P} \end{array}\right],$$

where *j_i_* are products of the B-spline basis functions, following from the definition (Rueckert et al., 1999). The derivative of the B-spline transformation is therefore a relatively small and sparse matrix, with repetitive elements, thus only *P* elements need to be computed instead of *d*^2^*P* or even *dN*. Again examining (3) we can see that the multiplication $J^{T}\frac{\partial I_{M}}{\partial x}$ can also be accelerated by omitting the empty parts.

Further optimizations to the source code resulted from a combination of Callgrind profiling and visual inspection of the source code, and include: (i) Allocated large vectors or matrices only once and re-use them throughout the registration. Examples include the cost function derivative ***g**_k_*, the transformation parameters **μ***_k_* and the transformation derivative ***J***, and in the optimizer the new position **μ**_*k* + 1_; (ii) Avoided repeated initializations of large arrays (fill with zeros), and additionally optimized this operation using std::fill (contributed back to ITKv4); (iii) Optimized some often used functions by avoiding ITK iterators, the use of loop unrolling, memcpy, etc; (iv) Compared to the previous implementation the amount of memory accesses were reduced when interpolating the moving image value and gradient; (v) Implemented gradient computation for the linear interpolator, which can compute the moving image gradient ∂ *I_M_* / ∂ ***x*** [see Equation (3)] much faster than the existing implementation of the first order B-spline interpolator; (vi) Made use of a new ‘scan line’ iterator from ITKv4 with low overhead.

### 3.2. GPU

For implementing algorithms on the GPU we have chosen to build on ITKv4's recent addition for GPU acceleration. This module wraps the OpenCL 1.2 API in an ITK-style API, while taking care of OpenCL initialization, program compilation, and kernel execution. It also provides convenience classes for interfacing with ITK image classes and filtering pipelines.

In the OpenCL design of ITKv4 important parts of the OpenCL specification were missing, most notably the queueing mechanisms and event objects. We implemented a large part of the OpenCL class diagram, where classes are responsible for a specific task conforming to the OpenCL standard. OpenCL event objects are used to synchronize execution of multiple kernels, in case a program consists of multiple kernels. We take advantage of the scheduling and synchronization mechanisms of OpenCL for the implementation of the GPU version of the resampler, see Section 3.2.2, where individual kernels have to be executed in order. In addition, we have added debugging and profiling functionality, which are useful features during development and for understanding performance bottlenecks of GPU architectures. A number of modifications have been made to improve design, implementation, and platform support (Intel, AMD, NVidia), thereby enhancing the existing ITKv4 GPU design.

We identified two independent registration components that allow for parallelism: the Gaussian pyramids and the resampling step. The Gaussian filtering relies on a line-by-line causal and anti-causal filtering, where all image scan lines can be independently processed; The resampling step requires for every voxel the same independent operation (transformation followed by interpolation).

#### 3.2.1. Pyramids

It is common to start the registration process (1) using images that have lower complexity, to increase the chance of successful registration. To this end images are smoothed and optionally downsampled, the latter either using linear interpolation (resampling) or by subsampling without interpolation (shrinking). The Gaussian pyramid is by far the most common one for image registration, and the computation of this pyramid we target to accelerate. The Gaussian filter computes infinite impulse response convolution with an approximation of the Gaussian kernel $G(x;\sigma )=\frac{1}{\sigma \sqrt{2\pi }}\exp\left(-x^{2}/2\sigma^{2}\right)$ (Deriche, 1990). This filter smoothes the image in a single direction only, and is therefore subsequently called for each direction to perform full smoothing.

The filter performs execution row-by-row for the direction *x* or column-by-column for the direction *y*, and similarly for direction *z*. All rows or columns can be processed independently, but columns can only be processed when all rows have finished. This execution model is therefore suitable for the GPU, by assigning each row or column to a different thread, which can then be executed in parallel. The column kernel is scheduled to start after the row kernel, using the OpenCL queues.

To achieve better performance each thread uses the local GPU memory, which is fastest, but this introduces a limitation on the input image size. Current GPUs usually only have 16kB of local memory, and the algorithm allocates three floating point buffers the size of the row/column (input, output plus temporary buffer). This results in a maximum image size of 1365 pixels, and therefore our GPU implementation works only for images of maximum size [1365,1365] or [1365,1365,1365]. This limitation can be avoided by using other platforms with a larger local memory (e.g., Intel CPUs allow 32kB), or by changing the algorithm altogether (e.g., by direct convolution with a truncated Gaussian kernel).

#### 3.2.2. Resampling

Resampling is the process of computing the value *I_M_*(***T***(***x***)) for every voxel ***x*** inside some domain. Usually, the fixed image domain Ω*_F_* is chosen, meaning that the computational complexity is linearly dependent on the number of voxels in the fixed image. The procedure is simple: 1) loop over all voxels ***x*** ∈ Ω*_F_*, 2) compute its mapped position ***y*** = ***T***(***x***), 3) obtain the moving image intensity *I_M_*(***y***) by interpolation, since ***y*** is generally a non-voxel position, and 4) copy this value to the output image.

Notice from above that the procedure is dependent on a choice of the interpolator and the transform. Several methods for interpolation exist, varying in quality and speed. Available implementations in elastix are nearest neighbor, linear and B-spline interpolation. There are also many flavors of transformations. The ones available in elastix in order of increasing flexibility, are the translation, the rigid, the similarity, the affine, the nonrigid B-spline and the nonrigid thin-plate-spline-like transformations, as well as arbitrary combinations of them by function composition, i.e., ***T***(***x***) = ***T**_n_*(… ***T***_2_(***T***_1_(***x***))). The latter is frequently used in image registration, for example when a rigid or affine registration is performed prior to a nonrigid B-spline registration.

**Figure 1:**
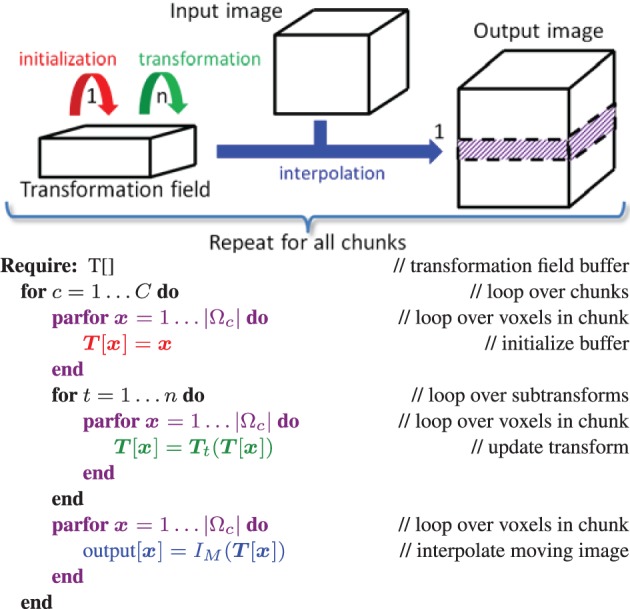
**Design of the resample filter on the GPU**. We select a chunk of the output image, initialize it (red kernel), and for that chunk a series of transformations ***T**_n_*(… ***T***_2_(***T***_1_(***x***))) are computed and stored in the intermediate transformation field (green kernels). After these transformation kernels have finished, the input image is interpolated and the result is stored in the output image chunk (blue kernel). Then we proceed to the next chunk. The loops in purple are computed in parallel.

In the ITK C++ implementation the flexibility to use any transformation in combination with any interpolator is achieved using classes and virtual methods. This flexibility introduces a major challenge when implementing a GPU version of the resampler. As mentioned earlier, OpenCL is a simplified C language specification, which does not provide a way of implementing virtuality on kernels, or the use of function pointers. In order to solve this issue, we propose to split the OpenCL kernel for the resampler in three groups of kernels, see also Figure 1:

Initialization: The first part is an OpenCL kernel responsible for the initialization of the deformation field buffer.
Transformation: This part consists of multiple OpenCL kernels each performing a single transformation ***T**_i_* sequentially.
Interpolation: The last part is an OpenCL kernel performing the interpolation *I_M_*(***T***(***x***)).

The OpenCL queueing mechanism utilizing OpenCL event lists, is employed for scheduling, to make sure that all kernels are executed successively. Within a kernel voxels are processed in parallel. A transformation field buffer is required to store the intermediate result of all sub-transformation kernels implementing ***T**_i_*. The resample kernel code is constructed from these multiple kernels during instantiation of the resample filter. Construction of all kernels is performed on the host (the CPU) at runtime. All initialization, transformation and interpolation kernels are sequentially scheduled on the target device (GPU) using the event list functionality. All kernels are provided with their arguments (inputs), such as input image, resampling domain, etc. The thus generated code is compiled for the GPU at runtime, and then executed. NVidia has implemented a mechanism to cache the compiled GPU binaries, thereby avoiding the need to re-compile the code after the first run. To be able to process large 3D images that may not fit on the GPU memory entirely, we additionally implemented a mechanism to process the input data in chunks, see Figure 1. While the input (*I_M_*) and output (*I_M_*(***T***)) images are loaded resp. allocated entirely, only a relatively small amount of memory is then needed for the intermediate transformation field. This buffer is reused until the full image is resampled.

GPU versions of all common transformations and interpolators were implemented, as well as arbitrary compositions of them.

### 3.3. Diagnostic classification of AD

The optimized registration procedure was validated with an experiment of classification of AD patients and healthy controls. The classification was based on two types of features, voxel-wise and region-wise features, which were extracted from structural MRI. These feature extraction approaches involve numerous image registrations steps, which were performed with both the accelerated version of elastix and the most recent release elastix v4.6. The classification performances were compared between the two versions, because then we can see in practice, in an application that makes heavy use of rigid and nonrigid registration, if and how much the results are affected by the acceleration. In this section the methods for the classification experiment are explained.

#### 3.3.1. Data

Data from the ADNI^1^ database was used. The ADNI cohort used for our experiments is adopted from the study of Cuingnet et al. (2011), from which we selected the AD patient group and the normal elderly control group. The inclusion criteria for participants were defined in the ADNI GO protocol (www.adni-info.org/Scientists/AboutADNI.aspx\#). The patient group consisted of 137 patients (67 males, age = 76.0 ± 7.3 years, Mini Mental State Examination (MMSE) score = 23.2 ± 2.0), and the control group of 162 participants (76 males, age = 76.3 ± 5.4 years, MMSE = 29.2 ± 1.0). The participants were randomly split into two groups of the same size, a training set and a test set, while preserving the age and sex distribution (Cuingnet et al., 2011). Structural MRI (T1w) data were acquired at 1.5T according to the ADNI acquisition protocol (Jack et al., 2008).

#### 3.3.2. Image processing

Tissue segmentations were obtained for GM, white matter (WM), and cerebrospinal fluid (CSF) using SPM8 (Statistical Parametric Mapping, London, UK). For estimation of intracranial volume, a brain mask was required for each subject. This brain mask was constructed using a multi-atlas segmentation approach using 30 atlases (see Section 3.3.3). We performed brain extraction (Smith, 2002) on the T1w images associated with the 30 atlases (Hammers et al., 2003; Gousias et al., 2008), checked the brain extractions visually, and adjusted extraction parameters if needed. The extracted brains were transformed to each subject's image and the labels were fused, resulting in a brain mask for each subject.

#### 3.3.3. Image registration: template space and ROI labeling

**Figure 2:**
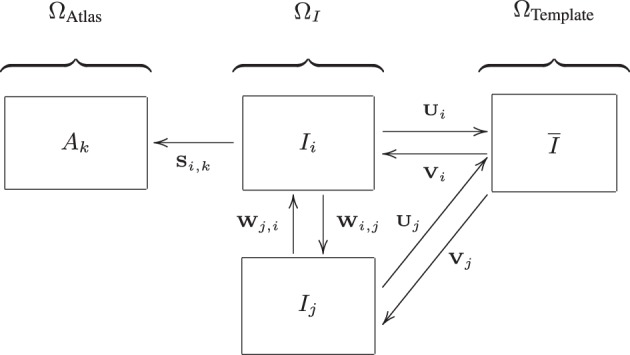
**Image spaces defined within the ADNI structural MRI data: image space (Ω*_I_*) and the template space (Ω_Template_)**. Another image space (Ω_Atlas_) is defined for the 30 atlas images. Transformations between the image spaces are indicated by **S**, **U**, **V**, and **W**. The arrows are pointing from the fixed to the moving domain. Different subjects are represented by *i* and *j*, the different atlas images are represented by *k*. From all *I_i_*, a template space image (*I*) is calculated (Section 3.3.3).

Voxel-wise features were extracted in a common template space (Ω_Template_, see Figure 2) based on the data of the training set. This common template space was constructed using a procedure that avoids bias toward any of the individual training images (Seghers et al., 2004). In this approach, the coordinate transformations from the template space to the subject's image space (**V***_i_*: Ω_Template_ → Ω*_I_i__*) were derived from pairwise image registrations. For computation of **V***_i_*, the image of an individual training subject (*I_i_*) was registered to all other training images (*I_j_*) using *I_i_* as the fixed image. This resulted in a set of transformations **W***_i,j_* : Ω_*I_i_*_→Ω_*I_j_*_. By averaging the transformations **W***_i, j_*, the transformation **U***_i_* : Ω_*I_i_*_→Ω_Template_ was calculated:

(5) $$U_{i}(x)=\frac{1}{N}\sum_{j=1}^{N}W_{i,j}(x).$$

The transformation **V***_i_* was calculated as an inversion of **U***_i_*: **V***_i_* = **U***_i_*^−1^. Note that the identity transformation **W***_i,i_* is also included in (5). The pairwise registrations were performed using a similarity (rigid plus isotropic scaling), affine, and nonrigid B-spline transformation model consecutively. The nonrigid B-spline registration used a three-level multi-resolution framework with isotropic control-point spacings of 24, 12, and 6 mm in the three resolutions respectively.

A template image was built using: $\bar{I}(x)=\frac{1}{N}\sum_{i=1}^{N}I_{i}(V_{i}(x)),$ with *I_i_*(**V***_i_*) representing the deformed individual training images. The test images were not included in the construction of Ω_Template_. For the test images, the transformation to template space (**V***_i_*) was obtained using the same procedure described above: using pairwise registration of each image with all training images, followed by averaging and inversion. Brain masks and tissue maps were transformed to template space using **V***_i_*.

**Figure 3:**
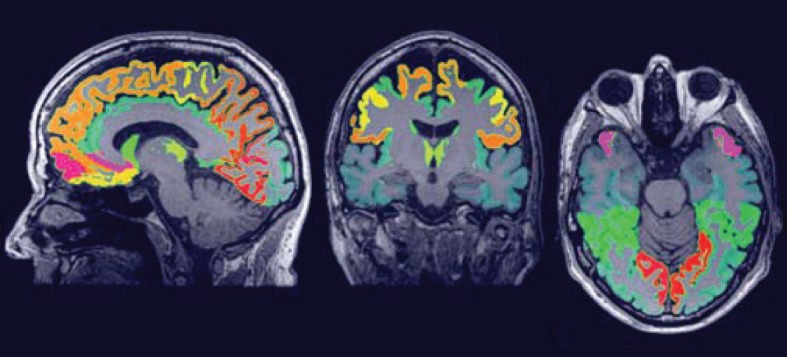
**The region labeling consisting of 72 ROIs in the brain**.

For extraction of the region-wise features, a set of 72 brain ROIs was defined for each subject individually in subject space (Ω*_I_*) using a multi-atlas segmentation procedure (Figure 3). Thirty labeled T1w images containing 83 ROIs each (Hammers et al., 2003; Gousias et al., 2008) were used as atlas images. The atlas images were registered to the subject's T1w image using a rigid, affine, and nonrigid B-spline transformation model consecutively resulting in transformation **S***_i,k_* : Ω*_I_i__* → Ω_Atlas*_k_*_. Registration was performed by maximization of mutual information within dilated brain masks (Smith, 2002). For initialization, the dilated brain masks were rigidly registered. For nonrigid registration, the same multi-resolution settings were used as in the template space construction. For this step, the subjects' images were corrected for inhomogeneities (Tustison et al., 2010). Labels were propagated to Ω_*I_i_*_ using **S***_i,k_* and fused using a majority voting algorithm (Heckemann et al., 2006). The brain stem, corpus callosum, third ventricle, lateral ventricles, cerebellum, and substantia nigra were excluded.

#### 3.3.4. Classification

Linear SVM classification was used with the LibSVM software package (Chang and Lin, 2011). Classification performance was assessed on the separate test set and quantified by the area under the receiver-operator characteristic curve (AUC). The SVM C-parameter was optimized using gridsearch on the training set.

Voxel-wise features were defined as GM probabilistic segmentations in the template space (Ω_Template_) (Klöppel et al., 2008; Cuingnet et al., 2011). A modulation step was performed, i.e., multiplication by the Jacobian determinant of the deformation field (Figure 2, transformation **V***_i_*), to take account of compression and expansion (Ashburner and Friston, 2000). This modulation step ensures that the overall GM volume was not changed by the transformation to template space.

The region-wise features were calculated in subject space (Ω*_I_*) as the GM volume in each ROI obtained from the probabilistic GM maps (Magnin et al., 2009; Cuingnet et al., 2011). To correct for head size, these features were divided by intracranial volume. All features were normalized to have zero mean and unit variance.

## 4. Experiments and results

### 4.1. Overview

For the evaluation we compare the accelerated implementations with the original implementations. Both runtime performance and accuracy are investigated.

To evaluate performance we compare the runtime per iteration between both algorithms, *t*_old_ and *t*_new_. The speedup factor is defined as 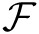 = *t*_old_ / *t*_new_. The speedup will depend on the number of threads *T* that are used for parallelization. The parallelization efficiency is a measure expressing how much a program is accelerated compared to an ideal speedup equal to the number of threads, i.e., ℰ = 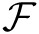 / *T*.

To evaluate accuracy we use a combination of measures, to make sure that the accelerated registration still returns similar results as the original. GPU pyramid and resampler results by OpenCL are compared with their original CPU version as a baseline, using the normalized root mean square error (nRMSE) as a measure of accuracy:

(6) $$\text{nRMSE}=\text{​​}\sqrt{\sum_{i=0}^{n}{\left(I_{\text{CPU}}(x_{i})\text{​}-\text{​}I_{\text{GPU}}(x_{i})\right)}^{2}/\sum_{i=0}^{n}I_{\text{CPU}}{\left(x_{i}\right)}^{2}}.$$

All timings were measured on a second run of the program, where the pre-compiled GPU kernel is loaded from cache. CPU optimizations were evaluated using the Alzheimer classification application to compare original with optimized methods, see Section 4.4.

While in our automatic testing environment (using CTest, part of the CMake package, www.cmake.org) we perform nightly evaluation on both 2D and 3D data, in this paper we only report 3D results. All timing experiments were run on a linux system, detailed in Table 1. This systems contains an NVidia GTX 480 graphical card (market launch March 2010), while currently (August 2013) the GTX 780 generation is available. All registrations for the diagnostic classification of AD were run on a cluster of linux systems.

**Table 1:** **Details of the system used for the timing tests**.

OS	Linux Ubuntu 12.04.2 LTS, 64 bit
CPU	Intel Xeon E5620, 8 cores @ 2.4 GHz
GPU	NVidia Geforce GTX 480
compiler	gcc 4.6.3
OpenCL	NVIDIA UNIX x86_64 Kernel Module 290.10

### 4.2. Parallelization and optimization on the CPU

CPU accelerations are evaluated by comparing the baseline algorithms with accelerated version, using various numbers of threads (*T* ∈ {1, 2, 3, 4, 8, 16}). We show registration results for the B-spline transformation, using a first order B-spline and a linear interpolator for the baseline and accelerated algorithms, respectively, with 3 resolutions and 1000 iterations per resolution. The B-spline grid is refined from the first to the last resolution, so that a progressively larger number of parameters *N* is used. In the experiments we inspect the influence of the number of samples |$\tilde{\Omega }$*_F_*| (2000 vs. 20,000), the B-spline grid spacing in the last resolution (10 mm vs. 5 mm, resulting in *N* = 2·10^3^, 9·10^3^, 5·10^4^ vs. *N* = 9·10^3^, 5·10^5^, 3·10^5^ parameters at each resolution, respectively), and the cost function (MSD vs. NC vs. MI).

**Figure 4:**
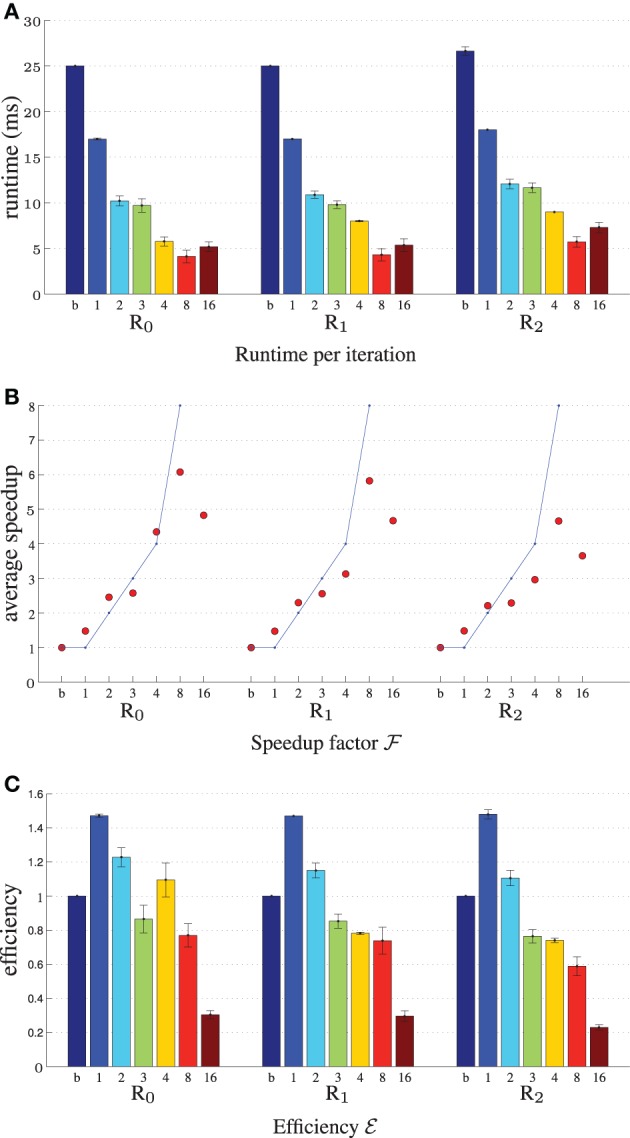
**Registration performance as a function of the number of threads**. R*_i_* denotes the resolution number, b refers to the baseline un-accelerated algorithm, and the numbers 1–16 refer to the number of threads used when running the parallel accelerated algorithm. The blue line shows ideal linear speedup. Results are shown for MI, *N* = 5·10^4^, |$\tilde{\Omega }$*_F_*| = 2000. **(A)** Shows the runtime per iteration, **(B)** the speedup factor 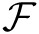, and **(C)** the efficiency **ℰ**.

Figure 4 displays the performance results for MI, 2000 samples, *N* = 5·10^4^, showing the reduction in runtime per iteration, the speedup factor and the parallelization efficiency. It can be seen that using more threads steadily increases the performance, until *T* matches the number of CPU cores. Further increasing parallelization decreases performance. The efficiency plot shows that although the performance increases with increasing *T*, the benefits are gradually diminished. An efficiency of 60–70% (Figure 4C) was obtained for 8 threads, which is influenced by the overhead of thread creation and destruction and by the fact that derivative joining (aggregating ***g**^t^_k_* to ***g**_k_*) is not free of cost. Comparing the columns “b” and “1” we can see that the general optimizations described in Section 3.1 already reduce runtime from 27 ms to 18 ms per iteration (R_2_), showing the overall benefits of these modifications. Separate tests used during development showed for example that computing ∂ *I_M_* / ∂ ***x*** using the linear interpolator instead of a first order B-spline was about 10–15x faster stand-alone, and using the new scan line iterator from ITKv4 when computing ***T***(***x***) for the B-spline transform was about 15% faster. Overall, the image registration was accelerated by a factor of 4–5x, when using 8 threads on our 8-core machine.

**Figure 5:**
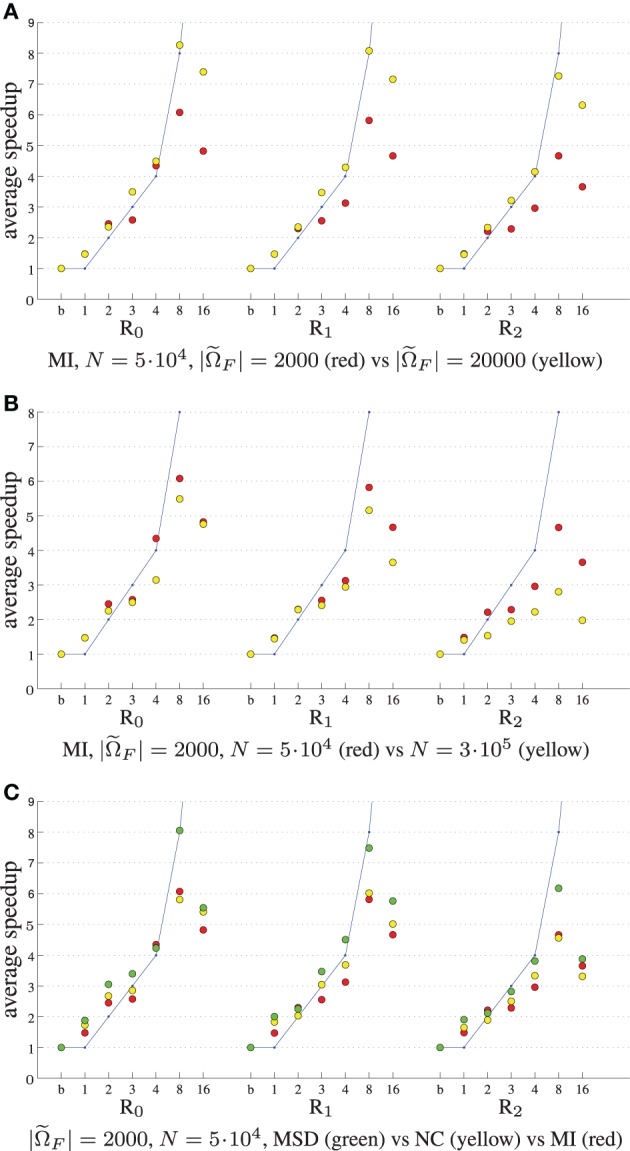
**Registration performance as a function of the number of threads**. R*_i_* denotes the resolution number, b refers to the baseline un-accelerated algorithm, and the numbers 1–16 refer to the number of threads used when running the parallel accelerated algorithm. The blue line shows ideal linear speedup. Varying the number of samples **(A)**, the number of registration parameters **(B)**, and the cost function **(C)**.

Figure 5 shows the experimental results when varying the number of samples |$\tilde{\Omega }$*_F_*|, parameters length *N* and cost function type. The speedup remains much closer to the theoretical limit when using 20,000 samples instead of 2000 (Figure 5A), although of course the former is 10 times as slow. This may be attributed to the fact that for many samples the overhead of thread creation and destruction is relatively small wrt computation time. In our current design we employ ITK's threading mechanism, which may be suboptimal for short tasks. Figure 5B shows that speedup decreases when the number of parameters is large (R_2_). In this case vector arithmetic [joining the derivatives ***g**^t^_k_* and performing the optimization step (2)] is starting to take a larger portion of an iteration. According to the Callgrind profiler about 15% of the time was spend for derivative joining and an additional ~7% for threading related initialization, and ~3% for the optimization step. In a separate test program we tested the performance of these operations comparing three versions: single threaded, multi-threaded using ITK and multi-threaded using OpenMP. We found that multi-threading was unsuccessful for the optimization step, only deteriorating performance, and successful for derivative joining, mostly so when using OpenMP. We therefore opted to only use multi-threading with OpenMP for the derivative joining. Finally, Figure 5C shows that all metrics almost equally well benefit from parallelization. Overall, the accelerations reduced the registration runtimes from 52, 57, and 80s to 10, 12, and 17 s for MSD, NC and MI, respectively (|$\tilde{\Omega }$*_F_*| = 2000, *N* = 5·10^4^), excluding optimization step size computation (~22s) of the ASGD optimizer.

### 4.3. Parallelization on the GPU

#### 4.3.1. Gaussian image pyramids

For testing the Gaussian pyramid accelerations we chose default scaling and smoothing schedules using 4 resolutions: images were downsized by a factor of 8, 4, 2, and 1 and smoothed with a Gaussian kernel with σ = 4, 2, 1 and 0 for the four resolutions, respectively. The results are shown in Table 2.

**Table 2:** **Results of the multi-resolution pyramid filter**.

**Image size**	**Resize**	*t***_CPU_**	*t***_GPU_**	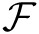	**nRMSE**
100 x 100 x 100	Off	0.05	0.02	2.3	0.55 × 10^−6^
	Resampler	0.06	0.03	1.9	0.52 × 10^−6^
	Shrinker	0.04	0.02	2.0	0.55 × 10^−6^
256 x 256 x 256	Off	0.84	0.33	2.5	0.56 × 10^−6^
	Resampler	0.98	0.58	1.7	0.52 × 10^−6^
	Shrinker	0.88	0.31	2.8	0.56 × 10^−6^
512 x 512 x 256	Off	4.07	2.51	1.6	0.57 × 10^−6^
	Resampler	4.68	2.19	2.1	0.53 × 10^−6^
	Shrinker	4.07	1.58	2.6	0.57 × 10^−6^

Timings shown are for all four levels in total.

The imprecision as measured by the nRMSE was quite small (<10^−6^), meaning that the the CPU and GPU returns almost exactly identical smoothed images. Small speedup factors of about two were measured, which may be an indication that the specific Gaussian smoothing algorithm is not very well suited for acceleration on the GPU.

#### 4.3.2. Image resampling

**Figure 6:**
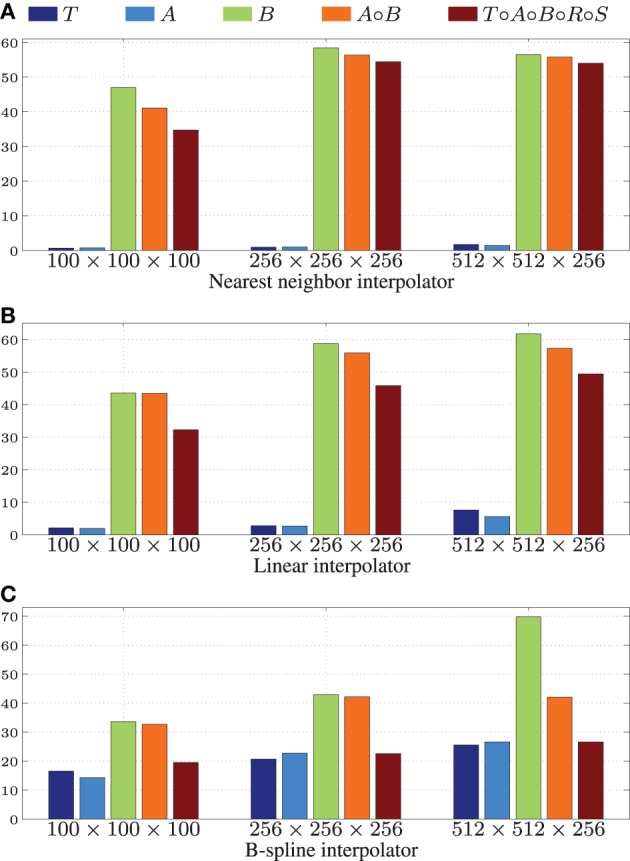
**Speedup factors** 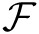 **for the GPU resampling framework**. Results are shown for the nearest neighbor **(A)**, the linear **(B)**, and the 3rd order B-spline interpolator **(C)**.

We tested the GPU resampling filter with different combinations of interpolators and transformations. For the B-spline interpolator and B-spline transform we have used third order splines. For brief notation we introduce the symbols *T*, *R*, *S*, *A* and *B* for the translation, rigid, similarity (rigid + isotropic scaling), affine and B-spline transformation, respectively. Detailed results are shown in Table 3 and Figure 6.

**Table 3:**
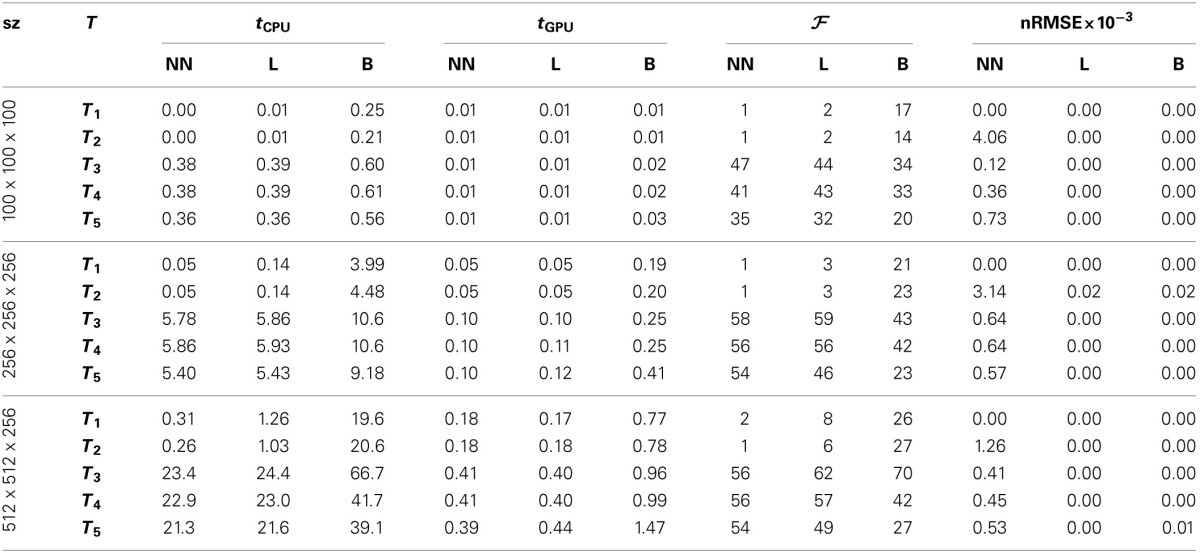
**Results of the resampling filter**.

Timings are shown in seconds. sz denotes image size. First, second and third number in each column denote the result for the nearest neighbor (NN), linear (L) and B-spline (B) interpolator, respectively. **T**_1_ − **T**_5_ are the composite transforms T, A, B, A ◦ B and T◦A◦B◦R◦S, respectively.

**Figure 7:**
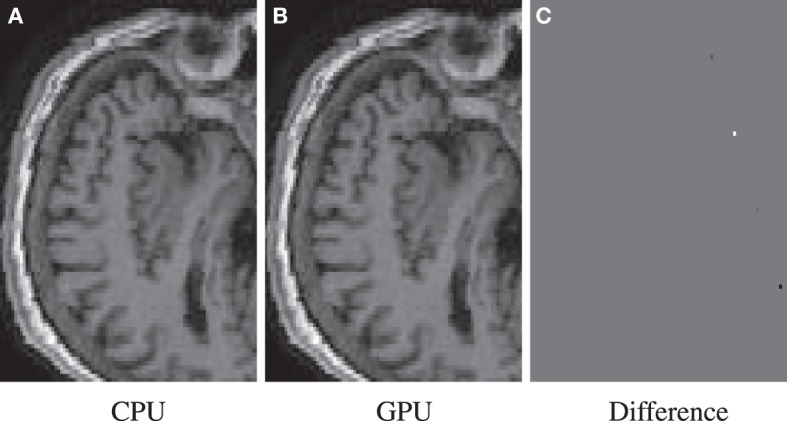
**Resample example for the highest nRMSE of Table 3 (NN, A, 100^3^)**. Differences are due to 79 isolated voxels in the range [−743, 502]. Shown are the result for the CPU **(A)**, the GPU **(B)**, and their difference **(C)**.

The GPU results for resampling were very close in terms of nRMSE to the output produced by the ITK CPU code. Only for the nearest neighbor interpolator in combination with the affine transformation higher errors are reported. This difference is due to floating point differences between CPU and GPU, sometimes leading to different rounding behavior. Example results are shown in Figure 7.

Figure 6 shows that linear transformations are accelerated less well than nonlinear transformations. This can be explained by (i) the small runtime of the linear transformations on the CPU, which is due to the CPU resampler implementing a highly optimized path for these cases, not possible for the GPU, and (ii) the lower computational complexity of these transformations (commonly more complex operations give more speedup on the GPU since GPU overhead is relatively small in those cases). Note that the B-spline interpolator yields higher speedup factors than the nearest neighbor and linear interpolator, for linear transformations (15–20 vs. 1–3), but lower speedup factors for nonrigid transformations (35–45 vs. 45–65). We remark that the reported speedup factors are a mixture of the speedup factors for the transformation and the interpolation step, related to the time spent in each step. For lower computationally complex transformations, the B-spline interpolator speedup will mostly determine the overall speedup, while for the more complex transformations both speedup factors determine the overall speedup. As a final observation, note the trend that more speedup is obtained for larger images, likely due to a better occupancy of the GPU combined with the copying overhead being less prominent in those cases.

Summarizing, speedups were obtained in the range 15–60x using more complex transformations, with no degradation for setups that were already very fast on the CPU. Using a B-spline interpolator and transform on a larger image, a common use-case, the execution time was 67 s on an 8 core CPU, while with a GPU this was reduced to <1 s.

### 4.4. Diagnostic classification of AD

#### 4.4.1. Registrations

**Figure 8:**
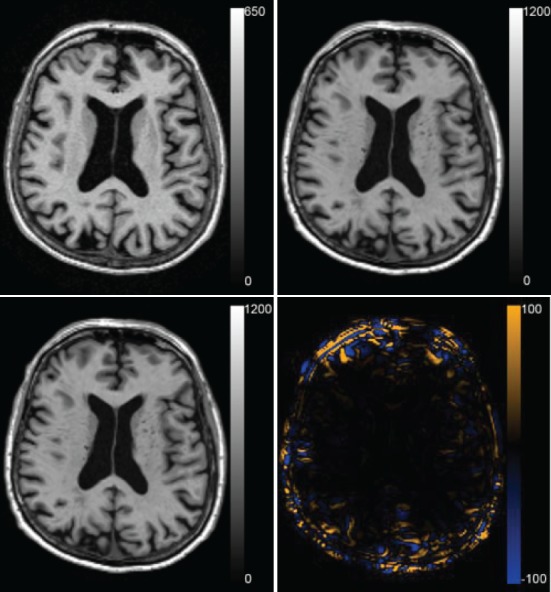
**Registration result for the median case of the voxel-wise method with a RMSE of 0.419 mm**. The fixed T1w image, the transformed moving T1w image registered with the original and the accelerated version of elastix and the difference between the two resulting images are shown.

To evaluate the registration results in the AD classification experiment, we compared the deformation fields obtained with the original and accelerated version of elastix. The RMSE between the two deformation fields was calculated. In the voxel-wise approach all 299 subjects' images were registered to the images of the 150 training subjects, which resulted in a mean ± std RMSE of the deformation field of 0.52 ± 0.46 mm (range: 0.0001–20.01 mm). In the region-wise approach 30 atlas T1w images were registered to all subjects' T1w scans. The RMSE was calculated in the same brain mask that was used for registration, which resulted in a RMSE of 0.75 ± 0.45 mm (range: 0.14–8.42 mm). The voxel sizes of the image is 0.95 × 0.95 × 1.20 mm^3^, so the average RMSE is smaller than the voxel dimension. Figure 8 shows an example of the registration with median RMSE for the voxel-wise approach. Registration time for the described setup reduced from ~13.1 to ~3.6 min per patient, of which optimization step size computation took 1.2 min.

#### 4.4.2. Features

**Figure 9:**
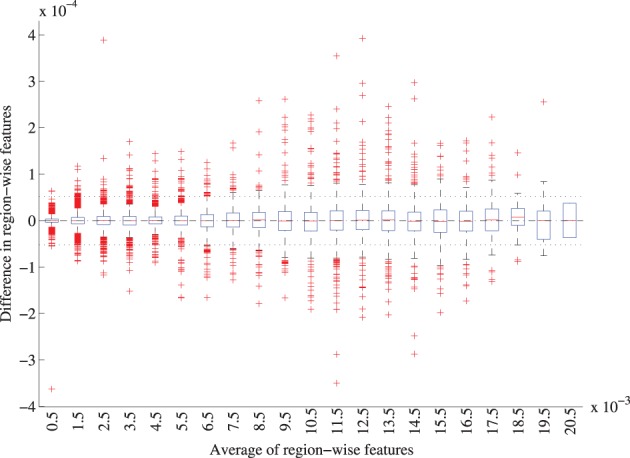
**Bland-Altman plot of the region-wise features for the original and accelerated versions of elastix**. The features represent the GM volume per brain ROI divided by the intracranial volume. The average features were grouped in bins of width 0.001, for each bin a boxplot is shown. 72 features for 299 subjects are included. The mean difference between the features is 1.0 · 10^−7^ (CI: −5.2· 10^−5^ ; 5.2· 10^−7^), mean and CI are indicated with the striped and dotted lined in the figure.

For the region-labeling, a high overlap was found between the ROIs using the two versions of the registration methods, resulting in a Dice coefficient of 0.97 ± 0.02 (mean ± std) over all ROIs in all subjects. Figure 9 shows a Bland-Altman plot for the region-wise features. The difference in the region volumes between the original and accelerated versions of the registration methods is very small compared to the mean.

**Figure 10:**
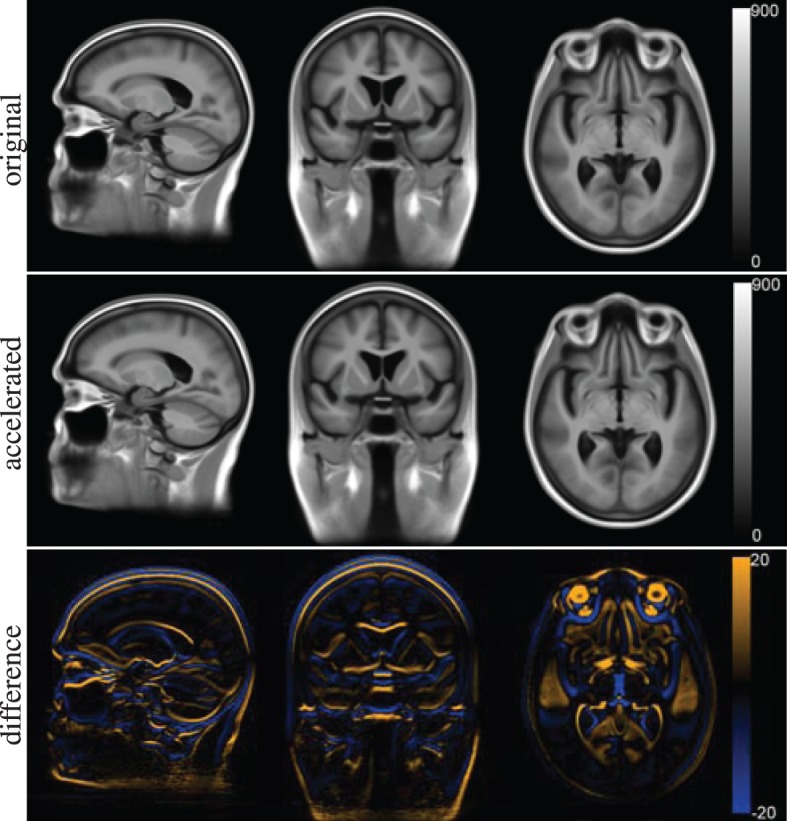
**Template space for the voxel-wise features constructed with the original version of elastix (top row) and the accelerated version (middle row)**. The difference between the two is shown at the **bottom** row.

The voxel-wise features cannot be compared directly as they are calculated in separate template spaces. Figure 10 shows the template spaces constructed with the original and accelerated version of the registration method. Although the template spaces show no visually observable differences, they do slightly differ (Figure 10C). The magnitude of the difference is much smaller than the magnitude of the template images. There seems to be a slight shift in the z-direction between the template spaces calculated with the two elastix versions.

#### 4.4.3. Classification performance

**Figure 11:**
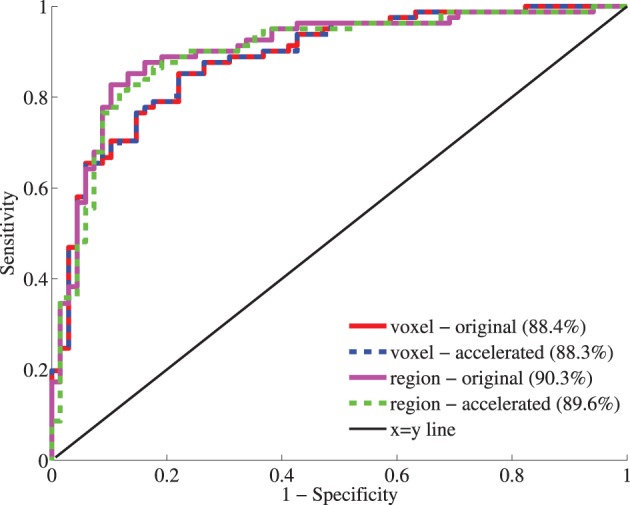
**Receiver-operator characteristic (ROC) curves for the classification based on voxel-wise (red, blue) and region-wise features (magenta, green) calculated with the original and accelerated versions of elastix**. Between brackets, the area under the curve (AUC) is given as performance measure.

Figure 11 shows the receiver-operator characteristic (ROC) curves for the classifications on the test set. The area under this curve (AUC) is a measure for classification performance. For the voxel-wise classifications, the features calculated with the original version of the registration software gave an AUC of 88.4%. The accelerated version resulted in a very similar AUC: 88.3%. For all test subjects (*n* = 149), the predicted labels were the same using both registration methods. For the region-wise method, performance was slighly better than for the voxel-wise method. Here, the original version resulted in a slightly higher AUC than the accelerated version (90.3% vs. 89.6%). Only three test subjects had a different prediction. To assess the difference between the two registrations methods, McNemar's binomial exact test was performed. For both voxel- and region-wise methods, the tests showed no significant difference (*p* = 1 in both cases).

## 5. Discussion and conclusion

In this paper we present a number of CPU and GPU optimizations for the image registration package elastix. The accelerated version of elastix was compared with the original in a study to automatically discriminate between AD patients and age- and gender-matched cognitively normal controls, based on T1w MRI.

Parallelization was used at several places of the image registration framework, exploiting the fork-and-join thread model of ITK, i.e., for computation of the cost function derivatives and for joining the results of the several threads. In addition, throughout the registration framework optimizations were performed, for example exploiting the sparseness of the derivative of the B-spline transformation, resulting in an overall increase in performance.

Compared to the original framework the optimizations only (no parallelization) accelerated image registration by 40–50%, see Figures 4, 5. Parallelization increases performance until the used number of threads reaches the number of CPU cores. We obtained an overall speedup of 4–5x, using 8 threads on an 8 core system. All registration similarity metrics almost equally well benefit from parallelization.

In addition to accelerating the core registration algorithm using the CPU, the GPU was used to accelerate two potentially computationally intensive components that are part of the algorithm. In this paper we accelerated computation of the multi-resolution Gaussian pyramid and the final resampling step, using OpenCL. A generic OpenCL framework was first developed, based on the existing ITKv4 GPU acceleration design. To this end a large part of the OpenCL specification was wrapped in ITK classes, following the OpenCL class diagram and inspired by current ITKv4 design. This generic architecture and close integration with ITK will ease adoption of OpenCL for general image processing tasks, not only for image registration. Subsequently, we designed a pipeline for pyramid computation and resampling, exploiting the design, notably the OpenCL queueing and synchronization mechanisms. The developed code is generic and allows extension to other geometric transformations and interpolators. The use of OpenCL furthermore enables targeting of most accelerator devices (GPU, FPGA) available today.

For the GPU optimizations speedup factors of ~2x were achieved for the image pyramids and 15–60x for the resampling, on larger images, using an NVidia Geforce GTX 480. For resampling, the increase in performance was negligible when using simple transformations (translation, affine) in combination with simple interpolators (nearest neighbor, linear), since in these cases the CPU computation was already quite fast (<1 s). For more complex operations (B-spline interpolator and/or B-spline transformation) the GPU is very beneficial.

To compare registration accuracy between original and accelerated versions of elastix, ~54k T1w image registrations have been performed with each version in the setting of an AD classification experiment. Registration results were similar as shown by visual inspection of the median result and the RMSE of the deformations field: 0.521 ± 0.460 mm (voxel-wise) and 0.749 ± 0.446 mm (region-wise). In addition, the classification features calculated with the two elastix versions were very similar. The differences in features between the two versions of the registration software were much smaller than the features themselves: for the voxel-wise approach the template spaces looked very similar, and for the region-wise approach the Dice overlap of the ROIs was very high and the differences between the GM volumes were relatively small. This resulted in a high classification performance, which was not significantly different between the two elastix versions.

Remaining differences between original and accelerated algorithms are attributed to a combination of algorithmic changes and hardware effects. For example, where in the original version the sample contributions (see Equation (3)) are directly accumulated in a single derivative, in the parallel version multiple derivatives are created, which are later joined to a single derivative. This changes the order and amount of arithmetic operations, and depending on machine precision this will lead to slightly different results. In addition, since image registration is an iterative process, small differences will be propagated until the end. In general, all implementation choices influence the final result. In the neuroimaging application the differences in the features (GM volumes) and classification results provide information on the impact of these imprecisions on the final result, which appears to be small.

Fast registration algorithms have most impact when used in a time-critical setting. An example would be the diagnostic classification of a single patient on a clinical workstation, performed by a neuro-radiologist. Generally, interactive speed is desired in such a user setting. The multiple registrations needed for the classification would be performed in parallel on a computing cluster, as was done in this work, which means that total classification time is limited by the runtime of a single registration. An example from outside the image-guided therapeutic domain would be (near) realtime motion compensation for radiation therapy. For research, fast registration enables testing of a wider range of algorithm parameters, or enables testing on large groups of patients within reasonable time. Given the general nature of similarity based image registration the results are naturally applicable to a wide range of image registration problems.

There are several areas in which our work can be improved and extended. For the CPU the total efficiency was 60–70% using 8 threads. When thread overhead is small compared to the computation, a much larger efficiency was obtained, see Figure 5A. This suggests that for short iteration times (5–6 ms, due to heavy stochastic subsampling during the optimization) the thread overhead is not negligible. The implementation of thread pools, that do not create and destruct threads every iteration, may mitigate this problem. Registration problems which need a high number of transformation parameters (large images and/or fine deformations) obtained only a small overall speedup (<3). In the current implementation the algorithmic steps related to vector arithmetics were found to be difficult to parallelize, and better methods have to be found. Further accelerations may be found by performing a comprehensive complexity analysis for each stage of the registration procedure, identifying the theoretical data transfer and computational requirements. Moreover, the use of more advanced profiling tools, such as the Intel VTune Amplifier, could be investigated. For the GPU we consider the use of pre-compiled binaries to completely remove compilation overhead at runtime. This functionally is available since the OpenCL 1.2 standard. Offloading of more parts of the registration algorithm to the GPU can also be considered. Considerable estimation time is still required by the ASGD optimizer (Klein et al., 2009b), which we addressed in separate work (Qiao et al., 2014).

The OpenCL implementation was additionally tested with an AMD Radeon HD 7900 card, and we can confirm portability of the solution. The AMD OpenCL compiler currently does not support caching of compiled binaries, making a timing comparison difficult. The CPU accelerations will be made available as open source in the next release of elastix. The GPU extensions are already incorporated in the elastix testing framework, but are not yet fully integrated in the elastix pyramids and resampler.

In conclusion, the proposed parallelization and optimizations substantially improve the runtime performance of image registration as implemented in the publicly available registration software elastix. This will facilitate medical practitioners and neuroimaging researchers, who commonly rely on image registration to label brain data, classify patients, compare between subjects or image sequences and to perform patient followup. It was shown in a large experiment on public data of patients with Alzheimer's disease that the registration results of the accelerated version are very close to the original. This work therefore makes substantially accelerated image registration accessible to a wide audience.

### Conflict of interest statement

The authors declare that the research was conducted in the absence of any commercial or financial relationships that could be construed as a potential conflict of interest.

## Footnotes

^1^The ADNI was launched in 2003 by the National Institute on Aging, the National Institute of Biomedical Imaging and Bioengineering, the Food and Drug Administration, private pharmaceutical companies and non-profit organizations, as a $60 million, 5-year public-private partnership. The primary goal of ADNI has been to test whether serial MRI, positron emission tomography (PET), other biological markers, and clinical and neuropsychological assessment can be combined to measure the progression of mild cognitive impairment (MCI) and early AD.
