# Supplementary material for: Fast parallel image registration on CPU and GPU for diagnostic classification of Alzheimer's disease
Source: Front Neuroinform. 2014 Jan 16;7:50. doi: 10.3389/fninf.2013.00050 (PMC3893567; doi:10.3389/fninf.2013.00050)
Supplement: Supplementary file 1 [file Presentation1.PDF]

Original Research Articles

Pages 1–2

Supplementary Material - Fast Parallel Image Registration on CPU and GPU for Diagnostic Classification of Alzheimer's Disease

Denis P. Shamonin [1], Esther E. Bron [2], Boudewijn P.F. Lelieveldt[1], Marion Smits[3], Stefan Klein[2], and Marius Staring [1,*], for the Alzheimer's Disease Neuroimaging Initiative[†]

[1] Leiden University Medical Center, Department of Radiology, Leiden, The Netherlands
[2] Erasmus MC, Biomedical Imaging Group Rotterdam, Departments of Medical Informatics and Radiology, Rotterdam, The Netherlands
[3] Erasmus MC, Department of Radiology, Rotterdam, The Netherlands

Table 1. Details of the system used for the timing tests.

OS	Windows 7, 64 bit
CPU	Intel Xeon E5-1560, 6 cores @ 3.2 GHz
GPU	NVidia Geforce GTX 780
compiler	Microsoft Visual Studio 2010
OpenCL version	OpenCL 1.1, CUDA 5.5, driver 320.57

1 INTRODUCTION

In the paper all performance results are obtained on an 8 core PC running at 2.4 GHz with an NVidia Geforce GTX 480 graphical card. In this supplement we provide results obtained on a modern system (late 2013), with 6 cores running at 3.2 GHz and an NVidia Geforce GTX 780 GPU. Details of this system are given in Table 1. Hyper-threading is enabled on this system, unlike on the system from the paper (older system).

2 RESULTS ON MODERN SYSTEM

2.1 Parallelization and optimization on the CPU

Results for the accelerations implemented on the CPU are given in Figure 1 and 2 for the modern system. They correspond to Figure 4 and 5 in the paper.

Figure 1 displays the performance results for MI, 2000 samples, $N = 5 \cdot 10^4$, showing the reduction in runtime per iteration, the speedup factor and the parallelization efficiency. It can be seen that using more threads steadily increases the performance, until T matches the number of CPU cores. Further increasing parallelization decreases performance. The efficiency plot shows that although the performance increases with increasing T, the benefits are gradually diminished. An efficiency of 80-90% (Figure 1c) was obtained for 6 threads, which is higher than the efficiency obtained on the older system (60-70% for 8 threads). Comparing the columns 'b' and '1' we can see that the general optimizations already reduce runtime from 21 ms to 15 ms per iteration (R_2). Overall, the image registration was accelerated by a factor of 5-6x.

Table 2. Results of the multi-resolution pyramid filter. Timings shown are for all four levels in total.

image size	resize	t_{CPU}	t_{GPU}	$\mathcal{F}$	nRMSE
100x100x100	off	0.04	0.02	1.8	0.69 $\times 10^{-6}$
	resampler	0.05	0.03	1.6	0.72 $\times 10^{-6}$
	shrinker	0.04	0.02	1.9	0.70 $\times 10^{-6}$
256x256x256	off	0.56	0.21	2.7	0.91 $\times 10^{-6}$
	resampler	0.69	0.32	2.2	0.91 $\times 10^{-6}$
	shrinker	0.50	0.21	2.4	0.91 $\times 10^{-6}$
512x512x256	off	2.75	0.87	3.2	0.75 $\times 10^{-6}$
	resampler	3.72	1.50	2.5	0.54 $\times 10^{-6}$
	shrinker	2.51	0.81	3.1	0.75 $\times 10^{-6}$

Figure 2 shows the experimental results when varying the number of samples $|\widetilde{\Omega}_F|$, parameters length N and cost function type. The speedup is higher when using 20000 samples instead of 2000 (Figure 2a), although of course the former is ten times as slow, like was seen on the older system. Unlike the older system, the modern system still benefitted from adding threads beyond the number of physical cores. This is attributed to the use of hyper-threading. Note that hyper-threading only showed beneficial for sufficiently long iteration times. Figure 2b shows that speedup decreases when the number of parameters is large (R_2), like the older system. Finally, Figure 2c shows that all metrics almost equally well benefit from parallelization.

Overall, the accelerations reduced the registration runtimes from about a minute to ∼10s ($|\widetilde{\Omega}_F| = 2000$, $N = 5 \cdot 10^4$), excluding optimization step size computation (15-20s) of the ASGD optimizer.

2.2 Parallelization on the GPU

* to whom correspondence should be addressed

† Data used in preparation of this article were obtained from the Alzheimer's Disease Neuroimaging Initiative (ADNI) database (adni.loni.ucla.edu). As such, the investigators within the ADNI contributed to the design and implementation of ADNI and/or provided data but did not participate in analysis or writing of this report. A complete listing of ADNI investigators can be found at: http://adni.loni.ucla.edu/wp-content/uploads/how_to_apply/ADNI_Acknowledgement_List.pdf

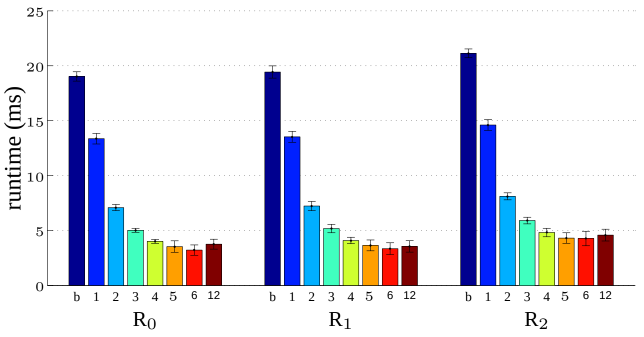

(a) Runtime per iteration

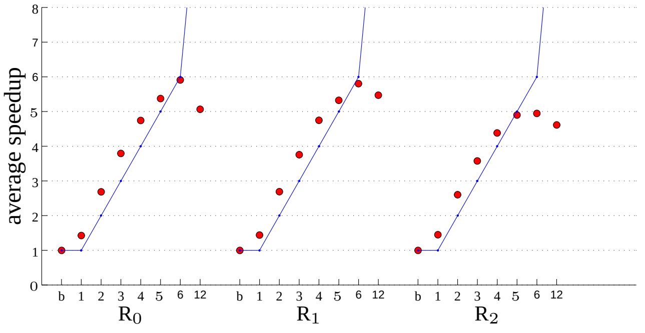

(b) Speedup factor $\mathcal{F}$

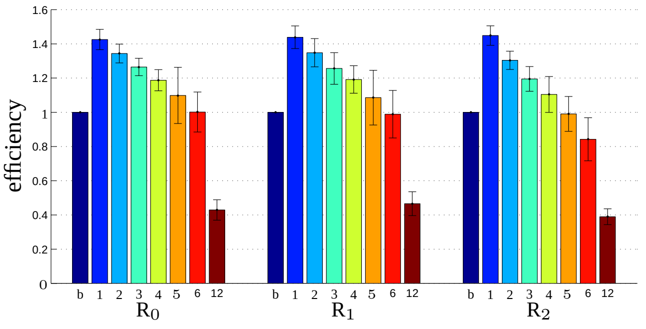

(c) Efficiency $\mathcal{E}$

Fig. 1. Registration performance as a function of the number of threads. R_i denotes the resolution number, b refers to the baseline un-accelerated algorithm, and the numbers 1 - 16 refer to the number of threads used when running the parallel accelerated algorithm. The blue line shows ideal linear speedup. Results are shown for MI, $N = 5{\cdot}10^4$, $|\widetilde{\Omega}_F| = 2000$.

2.2.1 Gaussian image pyramids The speedup factors and accuracy results for the Gaussian pyramid computation for the modern system are shown in Table 2. This table corresponds to Table 2 from the paper. The imprecision as measured by the nRMSE was quite small ($< 10^{-6}$), meaning that the the CPU and GPU returns almost exactly identical. Small speedup factors of about 2-3 were measured.

2.2.2 Image resampling Detailed results for the resampling step for the modern system are shown in Table 3 and Figure 3. They correspond to Table 3 and Figure 6 from the paper.

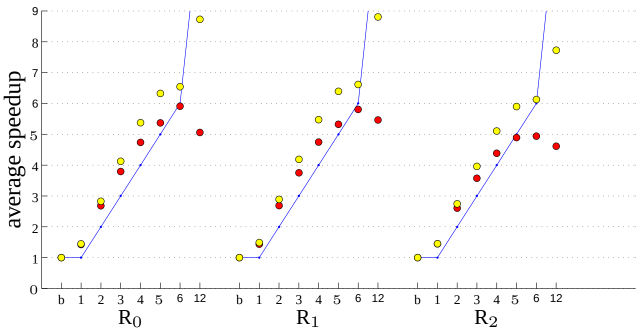

(a) MI, $N = 5{\cdot}10^4$, $|\widetilde{\Omega}_F| = 2000$ (red) vs $|\widetilde{\Omega}_F| = 20000$ (yellow)

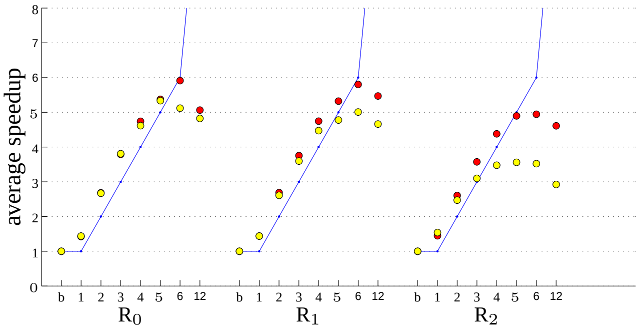

(b) MI, $|\widetilde{\Omega}_F| = 2000$, $N = 5{\cdot}10^4$ (red) vs $N = 3{\cdot}10^5$ (yellow)

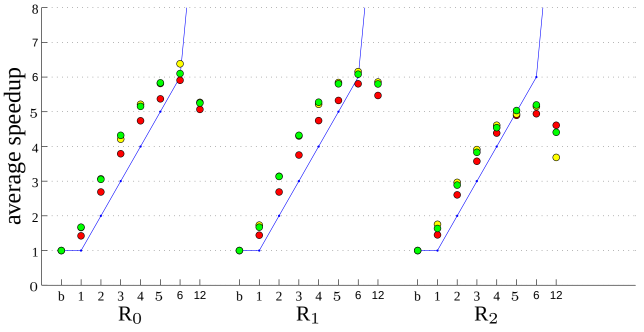

(c) $|\widetilde{\Omega}_F| = 2000$, $N = 5{\cdot}10^4$, MSD (green) vs NC (yellow) vs MI (red)

Fig. 2. Registration performance as a function of the number of threads. R_i denotes the resolution number, b refers to the baseline un-accelerated algorithm, and the numbers 1 - 16 refer to the number of threads used when running the parallel accelerated algorithm. The blue line shows ideal linear speedup.

The GPU results for resampling are the same in terms of nRMSE to the output produced by the ITK CPU code. There are no floating point differences on the modern NVidia GTX 780 graphical card, unlike reported on the older system. Speedups were obtained in the range 15 - 88x using more complex transformations. Using a B-spline interpolator and transform on a larger image, a common use-case, the execution time was 29 s on the 6 core CPU, while with the GPU this was reduced to <0.5 s.

Table 3. Results of the resampling filter. Timings are shown in seconds. sz denotes image size. First, second and third number in each column denote the result for the nearest neighbor (NN), linear (L) and B-spline (B) interpolator, respectively. $\boldsymbol{T}_1 - \boldsymbol{T}_5$ are the composite transforms T, A, B, $A \circ B$ and $T{\circ}A{\circ}B{\circ}R{\circ}S$, respectively.

sz	$\boldsymbol{T}$	t_{CPU}			t_{GPU}			$\mathcal{F}$			nRMSE$\times 10^{-3}$		
		NN	L	B	NN	L	B	NN	L	B	NN	L	B
100x100x100	$\boldsymbol{T}_1$	0.00	0.01	0.15	0.00	0.01	0.01	1	2	13	0.00	0.00	0.00
	$\boldsymbol{T}_2$	0.00	0.01	0.14	0.00	0.00	0.01	1	2	11	0.00	0.00	0.00
	$\boldsymbol{T}_3$	0.30	0.34	0.49	0.01	0.01	0.01	45	51	37	0.00	0.00	0.00
	$\boldsymbol{T}_4$	0.33	0.34	0.47	0.01	0.01	0.01	39	42	33	0.00	0.00	0.00
	$\boldsymbol{T}_5$	0.26	0.27	0.37	0.01	0.01	0.01	31	31	25	0.00	0.00	0.00
256x256x256	$\boldsymbol{T}_1$	0.06	0.14	2.55	0.03	0.03	0.11	2	4	24	0.00	0.00	0.00
	$\boldsymbol{T}_2$	0.07	0.18	2.46	0.03	0.03	0.09	2	6	26	0.00	0.00	0.00
	$\boldsymbol{T}_3$	4.79	4.89	7.28	0.06	0.06	0.12	80	81	59	0.00	0.00	0.00
	$\boldsymbol{T}_4$	5.20	5.11	7.19	0.06	0.06	0.13	88	81	57	0.00	0.00	0.00
	$\boldsymbol{T}_5$	3.78	3.87	5.85	0.06	0.06	0.12	65	64	48	0.00	0.00	0.00
512x512x256	$\boldsymbol{T}_1$	0.21	0.54	10.8	0.12	0.10	0.37	2	5	29	0.00	0.00	0.00
	$\boldsymbol{T}_2$	0.24	0.53	11.6	0.10	0.11	0.37	2	5	31	0.00	0.00	0.00
	$\boldsymbol{T}_3$	18.2	18.5	28.6	0.22	0.22	0.48	81	84	60	0.00	0.00	0.00
	$\boldsymbol{T}_4$	18.6	18.2	28.7	0.23	0.22	0.49	83	82	59	0.00	0.00	0.00
	$\boldsymbol{T}_5$	15.5	15.5	25.1	0.21	0.23	0.48	74	66	52	0.00	0.00	0.00

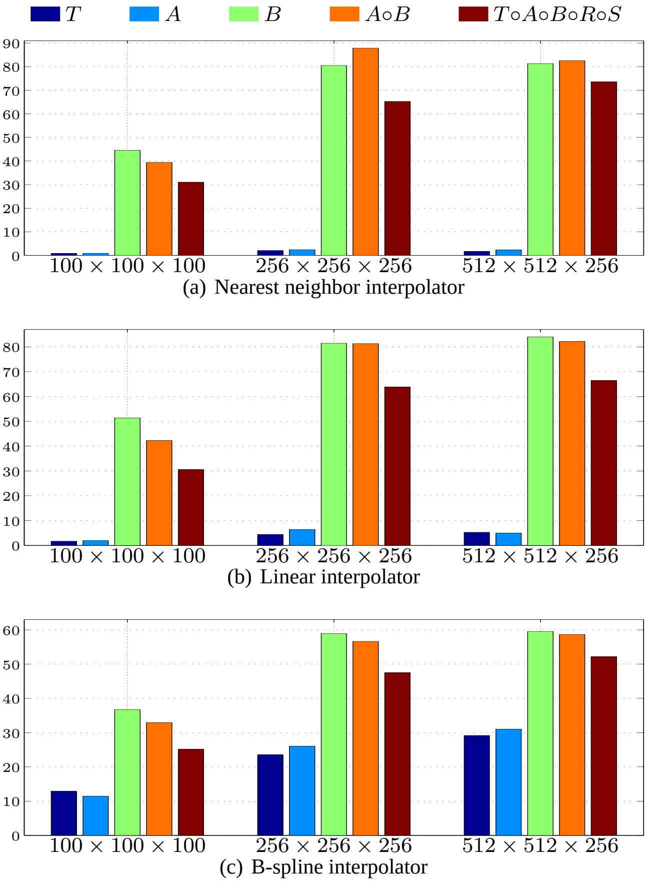

Fig. 3. Speedup factors $\mathcal{F}$ for the GPU resampling framework.
